# The Role of Aggregates of Therapeutic Protein Products in Immunogenicity: An Evaluation by Mathematical Modeling

**DOI:** 10.1155/2015/401956

**Published:** 2015-11-22

**Authors:** Liusong Yin, Xiaoying Chen, Abhinav Tiwari, Paolo Vicini, Timothy P. Hickling

**Affiliations:** ^1^Pharmacokinetics, Dynamics and Metabolism-New Biological Entities, Pfizer, Andover, MA 01810, USA; ^2^Pharmacokinetics, Dynamics and Metabolism-New Biological Entities, Pfizer, Cambridge, MA 02138, USA; ^3^Pharmacokinetics, Dynamics and Metabolism-New Biological Entities, Pfizer, San Diego, CA 92121, USA

## Abstract

Therapeutic protein products (TPP) have been widely used to treat a variety of human diseases, including cancer, hemophilia, and autoimmune diseases. However, TPP can induce unwanted immune responses that can impact both drug efficacy and patient safety. The presence of aggregates is of particular concern as they have been implicated in inducing both T cell-independent and T cell-dependent immune responses. We used mathematical modeling to evaluate several mechanisms through which aggregates of TPP could contribute to the development of immunogenicity. Modeling interactions between aggregates and B cell receptors demonstrated that aggregates are unlikely to induce T cell-independent immune responses by cross-linking B cell receptors because the amount of signal transducing complex that can form under physiologically relevant conditions is limited. We systematically evaluate the role of aggregates in inducing T cell-dependent immune responses using a recently developed multiscale mechanistic mathematical model. Our analysis indicates that aggregates could contribute to T cell-dependent immune response by inducing high affinity epitopes which may not be present in the nonaggregated TPP and/or by enhancing danger signals to break tolerance. In summary, our computational analysis is suggestive of novel insights into the mechanisms underlying aggregate-induced immunogenicity, which could be used to develop mitigation strategies.

## 1. Introduction

Therapeutic protein products (TPP) from nonhuman, humanized, and human origins include monoclonal antibodies (mAbs), Fc fusion proteins, blood factors, hormones, cytokines, chemokines, and engineered protein scaffolds [1]. They have been widely used to treat a variety of human diseases, including cancer, anemia, hemophilia, rheumatoid arthritis, multiple sclerosis, and inflammatory bowel diseases [1, 2]. Their large success is mainly due to increased target specificity, decreased intrinsic toxicity, and longer half-lives compared with small molecule drugs [3]. These advantages have led to the expansion of TPP in the drug market, with annual revenues of over 100 billion US dollars [1, 2]. However, unwanted immune responses against TPP, such as generation of anti-drug antibodies (ADA), have raised concerns on both drug efficacy and patient safety [4–8]. The effect of ADA on clinical outcomes ranges from no obvious impact to severe loss of efficacy and adverse effects such as infusion reactions [7]. The mechanisms leading to the generation of immunogenicity are yet to be established, but several risk factors have been proposed [9–12], which can be classified as follows: (i) patient-related: genetic background, immunological status, and prior exposure [10], (ii) treatment-related: route, dose, and frequency of administration [7, 13], and (iii) product-related: drug origins, characteristics such as protein structures and aggregates, and formulations [10].

**Figure 1:**
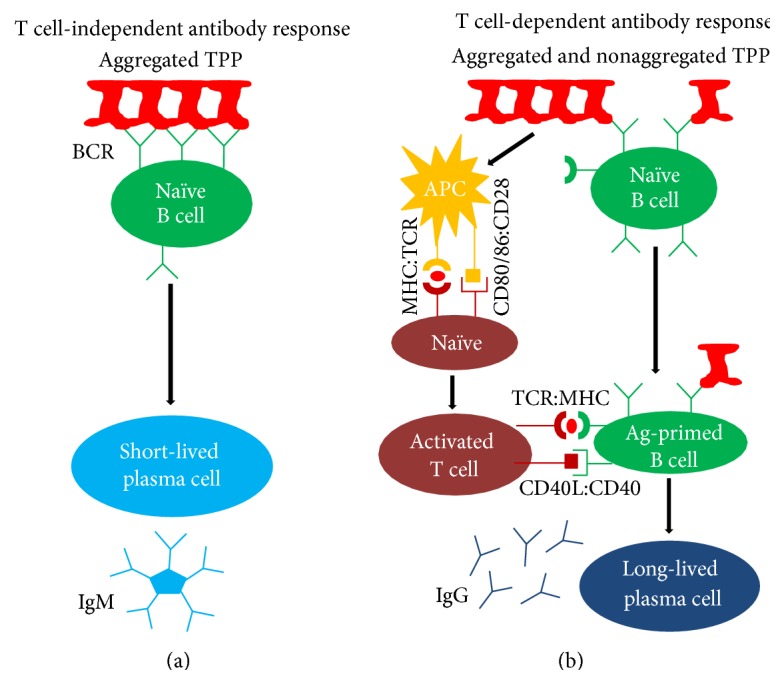
Schematic overview of aggregate-induced T cell-independent and T cell-dependent antibody responses. (a) In the T cell-independent pathway aggregates of TPP cross-link BCRs and activate B cells, which differentiate into short-lived plasma cells that generate antigen-specific IgM pentamers. (b) In the T cell-dependent pathway both aggregated and nonaggregated TPP can be captured by B cells or by APC which present TPP-derived epitopes to activate T cells, which in turn activate antigen-primed B cells. The activated B cells differentiate into long-lived plasma cells that generate isotype-switched IgG.

Among these risk factors, aggregates of TPP are of particular concern due to their potential role in inducing both T cell-independent and T cell-dependent immune responses [14–17] (Figure 1). It has been previously found that aggregated recombinant human interferon alpha2b generated by thermal stress, low pH, or oxidization stress is more immunogenic in mice compared with nonaggregated product [18–20]. High immunogenicity in mice has also been observed for aggregates of other TPP, such as human mAbs [21–23], human epoetin alfa [24], human factor VIII [25, 26], human interferon beta [27], and murine growth hormone [28]. In the clinic, the different ADA incidence rates for several recombinant human interferon beta drugs have been attributed to the differences in aggregation levels [29]. However, the detailed mechanism by which aggregates increase immunogenicity, especially in humans, is yet to be established. For example, it is unknown whether aggregates increase immunogenicity through a T cell-dependent or T cell-independent pathway; and which processes of ADA production could be altered by aggregates is also unknown. In the case of TPP, immunogenicity could be induced through both T cell-dependent and T cell-independent pathways [9, 12]. In the T cell-dependent pathway, antigenic peptides derived from TPP could be presented by major histocompatibility complex class II molecules (MHC II) on antigen-presenting cells (APC) that have been matured by danger signal to stimulate antigen-specific CD4+ T cells. Activated CD4+ T cells would then stimulate antigen-specific B cells that will be responsible for the production of ADA, which are usually affinity matured IgG. It has been found that, in comparison with the nonaggregated form, aggregated mAb results in an increase in the amount of total peptides and the number of epitopes eluted from MHC II [30]. This suggests that aggregates may increase immunogenicity by enhancing antigen processing and presentation in the T cell-dependent pathway. Aggregates could also contribute to T cell-dependent immunogenicity by increasing the danger signal for dendritic cell maturation. Consistent with this, a recent study suggested that aggregated mAb induces significantly higher dendritic cell maturation compared with unstressed mAb [30]. Lastly, aggregates could form repetitively arranged B cell epitopes in a paracrystalline manner to cross-link B cell receptors (BCRs), which in turn will activate antigen-specific B cells to generate ADA, mostly IgM, via the T cell-independent pathway [14]. However, the scarcity of clinical data and the difficulty to isolate the impact of aggregates from other immunogenicity risk factors are major impediments to understand the mechanisms of aggregate-induced immunogenicity.

Mathematical modeling offers the advantage of fast and cost-effective assessment and so it can be used in complement with experimental analysis to study immune responses [31–34]. It also provides quantitative means to dissect each component of a complex response for a deeper understanding of the mechanisms underlying aggregate-induced immunogenicity. Multiple mechanistic mathematical models have been previously developed to study immune responses against various pathogens. For example, antigen processing and presentation by APC and the activation of T helper cells by interactions between T cell receptors and MHC II-peptide complexes have been modeled and the simulation results agree with a variety of experimental data [35]. A mathematical model was also developed for predicting the clonal selection of B cells and antibody production by plasma cells [36]. The role of activation threshold and infections in the dynamics of autoimmune diseases has been studied mathematically as well [37, 38]. Mathematical models have been proposed and experimentally validated for T cell-dependent antibody responses to a wide range of antigens, including* Haemophilus influenzae* type b, hepatitis B virus, cancer antigens, and influenza A virus [39–43]. The T cell-independent activation of B cells by multivalent hapten-polymer has been modeled, where fitting to experimental data revealed that a minimum number of BCRs, in the range of 7 to 15, need to be cross-linked by a single multivalent ligand to stimulate a B cell [44, 45]. With regards to TPP-induced immunogenicity, several pharmacokinetics (PK) models have been developed to study the impact of ADA on mAb therapy [32]. For example, by incorporating ADA-drug interactions into empirical PK modeling, we developed a PK/ADA model to quantitatively assess the extent and timing of ADA generation, affinity maturation, and ADA-mediated TPP elimination [46]. More recently, we built a mechanistic, multiscale mathematical model of TPP-induced immunogenicity, recapitulating the key processes underlying T cell-dependent generation of ADA, such as antigen presentation, activation of immune cells, and production of ADA as well as* in vivo* disposition of ADA and TPP [47, 48]. This system-level model consists of a subcellular module for antigen presentation, a cellular module for immune system activation and antibody production, and a whole-body module for drug disposition. The model is able to reproduce key immunological phenomena such as antibody affinity maturation and enhanced secondary response [47, 48]. More importantly, a case study on immune response against adalimumab (a fully human anti-TNF alpha IgG1 mAb) showed reasonable agreement between model simulations and experimental observations [47, 48]. Owing to its flexibility and comprehensiveness this system-level model provides us with an ideal platform to probe mechanisms through which aggregates could generate immunogenicity.

In this study, we evaluate whether aggregates could induce T cell-independent or T cell-dependent immune response. In the former case, we model the interactions between multivalent aggregates and BCRs and examine the formation of signal-transducing complex (STC) under physiologically relevant conditions. For the latter case, we use our previously developed system-level model to investigate the impact of antigen processing and presentation, number and affinity of epitopes, and danger signal on ADA production due to aggregates.

## 2. Materials and Methods

### 2.1. Aggregates in the T Cell-Independent Pathway: Interactions between Multivalent Aggregates and BCRs

**Figure 2:**
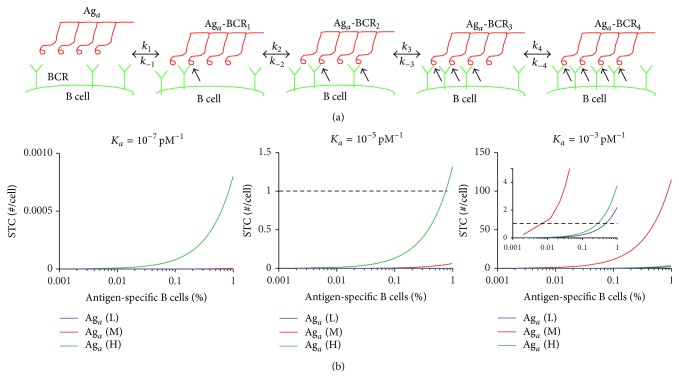
Significant number of STC per cell only forms under limited conditions. (a) Schematic representation of a tetravalent aggregate (Ag_*a*_) binding to BCRs to form Ag_*a*_BCR_*i*_, where *i* denotes the number of BCRs bound to Ag_*a*_. Black arrow points out the binding of Ag_*a*_ to a BCR. Each binding step *i* is governed by its binding (*k*_*i*_) and dissociation (*k*_−*i*_) rates. (b) Simulated levels of STC formed per cell are plotted against percentage of antigen-specific B cells under low (L, 0.1 pM), medium (M, 12 pM), and high (H, 1500 pM) levels of total Ag_*a*_, for binding affinity *K*_*a*_ = 10^−7^ pM^−1^ (left panel), 10^−5^ pM^−1^ (middle panel), and 10^−3^ pM^−1^ (right panel). Inset in the right panel is the zoomed-in version of the plot. STC per B cell is defined as the number of aggregates that cross-link a minimum number (*s*) of BCRs. Here *s* = 2 and valency *n* = 100. The horizontal dashed line denotes one STC.

An aggregate (Ag_*a*_) is assumed to be a homogeneous product formed by the combination of *n* monomers, which gives it a valency of *n*. The binding of Ag_*a*_ to BCR is assumed to be sequential (see Figure 2(a) for an example with *n* = 4) and can be represented by the following second-order reactions:

(1) $$\begin{array}{c} {\text{Ag}}_{a}+\text{BCR}\frac{k_{1}}{k_{-1}}{\text{Ag}}_{a}{\text{BCR}}_{1}\\ {\text{Ag}}_{a}{\text{BCR}}_{1}+\text{BCR}\frac{k_{2}}{k_{-2}}{\text{Ag}}_{a}{\text{BCR}}_{2}\\ \vdots \\ {\text{Ag}}_{a}{\text{BCR}}_{n-1}+\text{BCR}\frac{k_{n}}{k_{-n}}{\text{Ag}}_{a}{\text{BCR}}_{n}, \end{array}$$

where *k*_*i*_ and *k*_−*i*_ are the *i*th reaction's binding and dissociation rates, respectively, and Ag_*a*_BCR_*i*_ is the complex formed by binding of Ag_*a*_ to *i* BCRs. It is assumed that a BCR could bind to any free site on Ag_*a*_ and dissociate from any bound site on Ag_*a*_BCR_*i*_. The above reactions can be described by the following ordinary differential equations that govern the time evolution of Ag_*a*_BCR_*i*_, Ag_*a*_, and BCR:

(2) dAgaBCR1dt=n·k1·BCR·Aga+2·k−2·AgaBCR2−k−1+n−1·k2·BCR·AgaBCR1dAgaBCRidt=n−i+1·ki·BCR·AgaBCRi−1+i+1·k−i+1·AgaBCRi+1−ik−i+n−i·ki+1·BCR·AgaBCRi,1≤i≤n−1dAgaBCRndt=kn·BCR·AgaBCRn−1−n·k−n·AgaBCRndAgadt=−k1·n·BCR·Aga+k−1·AgaBCR1dBCRdt=−k1·n·BCR·Aga+k−1·AgaBCR1−∑j=2nkj·n−j+1·BCR·AgaBCRj−1+k−j·j·AgaBCRj.

We selected three (low, medium, and high) physiologically relevant levels for input parameters association constant (*K*_*a*_ = *k*_1_/*k*_−1_) and initial Ag_*a*_ concentration ([Ag_*a*_^0^]). [Ag_*a*_^0^] is Ag_*a*_ concentration at *t* = 0, as an initial condition for ordinary differential equations, which is estimated using the following equation:

(3) $$\begin{array}{c} \left[\;{{\text{Ag}}_{a}}^{0}\;\right]=\left[\;\text{Ag}\;\right]\cdot \frac{p}{n}, \end{array}$$

where [Ag] is the total TPP concentration, *p* is the aggregation percentage in TPP, and *n* is the valency of aggregates. [Ag] ranges from 500 to 10^5^ pM based on 30 *μ*g dose of interferon beta 1b and 40 mg dose of anti-TNF mAb adalimumab, respectively [29, 47–49]; *p* spans from 2 to 15% based on a previous report on the characterization and quantitation of aggregates in recombinant human interferon beta drug products [29]; and *n* varies from 10 to 100 based on the sizes of nonaggregated and aggregated TPP [18, 23, 29, 50, 51]. Taken together, the low and high levels of Ag_*a*_^0^ are 0.1 and 1500 pM, respectively. The association constant *K*_*a*_ has been previously reported to be 10^−7^ pM^−1^ for antibodies with low intrinsic affinities and 10^−3^ pM^−1^ for affinity matured antibodies, and hence these were selected as low and high levels [52]. The middle levels for total Ag_*a*_^0^ (12 pM) and *K*_*a*_ (10^−5^ pM^−1^) are the geometric means of corresponding low and high levels. The rate of binding of an antigen to its corresponding BCR, *k*_*i*_, is relatively constant [52, 53], so we fixed it to 8.64 × 10^−3^ pM^−1^ day^−1^. By contrast, the rate of dissociation (*k*_−*i*_) is expected to increase with *i* because the resistance of Ag_*a*_ against torsion and bending grows due to the steric hindrance from progressive binding of BCRs [45]. For simplicity we assume that *k*_−*i*_ decreases exponentially with *i* and the base for exponential decay is 0.5 as previously identified while modeling interactions between multivalent hapten-polymer and BCRs [45]. The initial BCR concentration is the product of number of BCRs per cell, B cell concentration, and percentage of antigen-specific B cells. The number of BCRs per cell and B cell concentration have been previously reported as ~10^5^ and ~10^8^ L^−1^, respectively [41, 44, 45, 47, 48]. Studies on the percentage of antigen-specific B cells are limited, but it has been reported to be <0.002% for vaccinia virus [54] and <1% for individual antigens [55]. The above estimates were used to define the input range of BCR concentration at *t* = 0 as an initial condition for the ordinary differential equations in the simulation.

In the model, the STC is the number of Ag_*a*_ that cross-links at least *s* BCRs as defined in [44, 45]:

(4) $$\begin{array}{c} \text{STC}=\overset{n}{\underset{s}{\sum }}{\text{Ag}}_{a}{\text{BCR}}_{s}. \end{array}$$

The model was simulated using the ordinary differential equation solver* ode15s* in MATLAB (The MathWorks, Inc., Natick, MA).

### 2.2. Aggregates in the T Cell-Dependent Pathway: Impact on Antigen Processing and Presentation and Danger Signal

**Figure 3:**
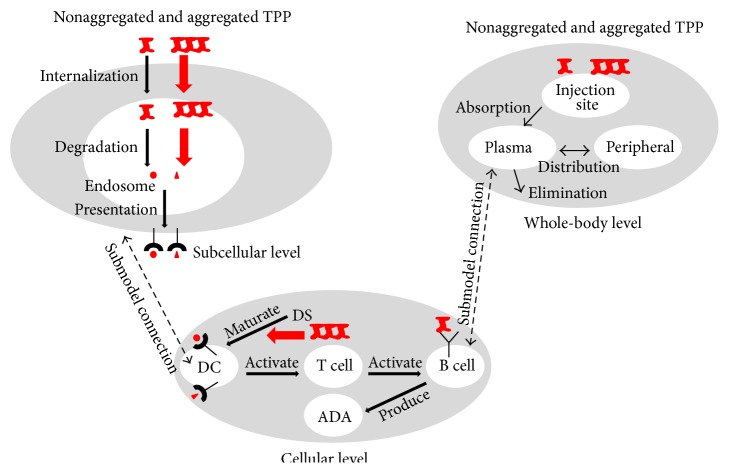
Schematic highlighting of the potential role of aggregates in T cell-dependent ADA production. A recapitulation of our system-level model for T cell-dependent ADA production [47, 48]. At the subcellular level, TPP are internalized into endosome of APC, such as dendritic cells (DC), and then degraded into antigenic peptides. Epitopes derived from TPP could be loaded onto MHC II and presented on the surface of APC. Aggregates could contribute to enhanced ADA production by having increased internalization or degradation rate or number and affinity of epitopes generated (indicated by thick red arrows). At the cellular level, danger signal (DS) maturated DC activate T cells which in turn activate B cells to generate ADA. Aggregates could enhance the DS to maturate DC (see red arrow). At the whole-body level, aggregated and nonaggregated TPP are absorbed from the injection site into plasma and will be distributed into periphery, eliminated, or captured by B cells through BCR binding.

For this analysis, we use our previously developed mechanistic, multiscale mathematical model for T cell-dependent ADA production [47, 48]. In this system-level model aggregates could contribute to increased ADA production by enhancing either the antigen processing and presentation or the danger signal for dendritic cell maturation (denoted by red arrows in Figure 3). We simulate the impact of aggregates by increasing (i) the rate of internalization of TPP into the endosome, (ii) the rate of degradation/processing of TPP into antigenic peptides, (iii) the number of epitopes generated, (iv) the affinity of epitopes to MHC II, and (v) the level of danger signal. Subsequently, for each of these conditions, we examine the endosomal levels of aggregates and epitope, the number of MHC II-peptide complexes on APC, and the levels of ADA production. To simulate B cell clonal selection and antibody affinity maturation, B cells and ADA are divided into 17 subgroups based on the binding affinity to antigen [36, 47, 48]. In our analysis, we define ADA production as the sum of the 17 subgroups.

## 3. Results

### 3.1. Aggregates Are Unlikely to Induce T Cell-Independent Immune Response because the Number of STC Formed Is Limited

To evaluate whether aggregates could induce T cell-independent antibody responses through BCR cross-linking, we examine the number of STC formed per B cell for different parameter combinations (see Section 2 for details). The model output for interactions between aggregates and BCR is the STC formed per B cell, which was previously defined as the number of Ag_*a*_ which cross-links a minimum number of BCRs [44, 45]. It has been reported that a multivalent ligand stimulates B cell activation only if it cross-links a minimum number (*s*) of BCRs, which is usually between 7 and 15 [44, 45]. We calculated the number of STC for *s* = 2, 5, and 10 under different total Ag_*a*_, *K*_*a*_, and BCR levels. Surprisingly, our computer simulation analysis showed that if *s* = 10 or 5, no more than one STC per cell could be observed under physiological levels of total Ag_*a*_, BCR, and *K*_*a*_ (data not shown). Even if *s* is lowered to 2, more than one STC per cell can form only under limited conditions, when the sensitive parameters are near the upper limits of the physiologically plausible ranges (Figure 2(b)). In the case of *K*_*a*_ = 10^−7^ pM^−1^, no more than one STC could form (Figure 2(b), left panel). For *K*_*a*_ = 10^−5^ pM^−1^, more than one STC could form at high levels of total Ag_*a*_ (1.5 × 10^−3^ pM) but only near the upper limit of antigen-specific B cells percentage (1%) (Figure 2(b), middle panel). Finally, when *K*_*a*_ = 10^−3^ pM^−1^, more than one STC could form at all total Ag_*a*_ levels but only with antigen-specific B cell percentage >0.006% (Figure 2(b), right panel). These results from our computer simulation showed that STC per cell is very sensitive to *K*_*a*_ and total concentrations of Ag_*a*_ and BCRs (but not to binding rate *k*_*i*_, data not shown). Overall, this analysis suggests that aggregates are unlikely to induce T cell-independent activation of B cells and consequent ADA production under physiologically plausible conditions. Therefore, aggregates may only contribute to ADA production through a T cell-dependent pathway, which we explore next.

### 3.2. Aggregates Could Enhance ADA Production by Increasing the Danger Signal to Maturate Dendritic Cells

**Figure 4:**
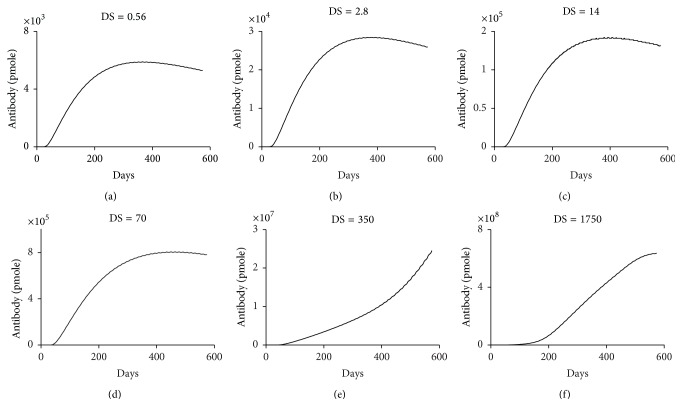
Aggregates could contribute to ADA production by increasing danger signal to maturate dendritic cells. ((a)–(f)) Simulated ADA production is shown at various levels of danger signal (DS) which is modeled as the amount of LPS in ng. Remaining parameter values are the same as in the original simulation for nonaggregated adalimumab [47, 48]. DS = 350 ng LPS shows the original simulation for nonaggregated adalimumab [47, 48]. ADA production is shown as the sum of the 17 subgroups. Dose = 40 mg administered biweekly.

To evaluate the T cell-dependent effect of aggregates on ADA production, we modulated those parameters in our system-level immunogenicity model [47, 48] that may be impacted by aggregation. This model consists of a subcellular module for antigen presentation in APC, a cellular module for immune cell activation and ADA production, and a whole-body module for drug and ADA disposition (Figure 3). Aggregates have been previously shown to increase danger signal for dendritic cell maturation and T cell activation [12, 22, 30, 56]. Specifically, aggregated mAb upregulated the dendritic cell maturation marker CD83 and CD4+ T cell costimulatory molecules CD80 and CD86 as well as cytokines produced by CD4+ T cells, such as IL-2 and IL-10 [30, 56]. Due to the complexity of dendritic cell maturation by danger signal and the unavailability of many parameters associated with this process, it is simply modeled as being driven by endotoxin lipopolysaccharide (LPS) [47, 48]. LPS is widely used in immunological studies for dendritic cell maturation [57–61] and is present in many TPP [62]. The cytokine profiles induced by LPS and aggregates of mAb are very similar [22, 63]. Using our system-level model, we previously simulated ADA production induced by adalimumab, a fully anti-TNF alpha IgG1 mAb used to treat various inflammatory and autoimmune diseases, with a danger signal of 350 ng LPS [47] (Figure 4(e)). If aggregates increase the danger signal by 5-fold, ADA production is increased by 20-fold (Figure 4(f)). We also simulated ADA production for low danger signal levels (Figures 4(a)–4(d)) as the actual amount induced by nonaggregated TPP is unknown. In essence, ADA production depends on the level of danger signal (Figures 4(a)–4(f)). Therefore, our simulations suggest aggregates could enhance ADA production by increasing danger signal to enhance maturation of dendritic cells and subsequently activate T cells.

### 3.3. Aggregates Could Not Enhance ADA Production by Increasing Antigen Processing and Presentation If High Affinity Epitopes Are Already Present in Nonaggregated TPP

Antigen processing and presentation are the key events in T cell-dependent immunogenicity of TPP [12]. Previous studies have demonstrated that aggregation enhances antigen's uptake, processing, and presentation by APC [12, 22, 30, 56, 64]. More recently, a study showed that aggregated mAb could directly increase the total number of different peptides and the number of epitopes presented by MHC II compared with nonaggregated mAb [30]. To evaluate whether aggregation-enhanced antigen processing and presentation could increase ADA production, we simulated these effects of aggregates in our model by changing its internalization or degradation rate or the number and affinity of epitopes generated and assessing their impact on final ADA production.

**Figure 5:**
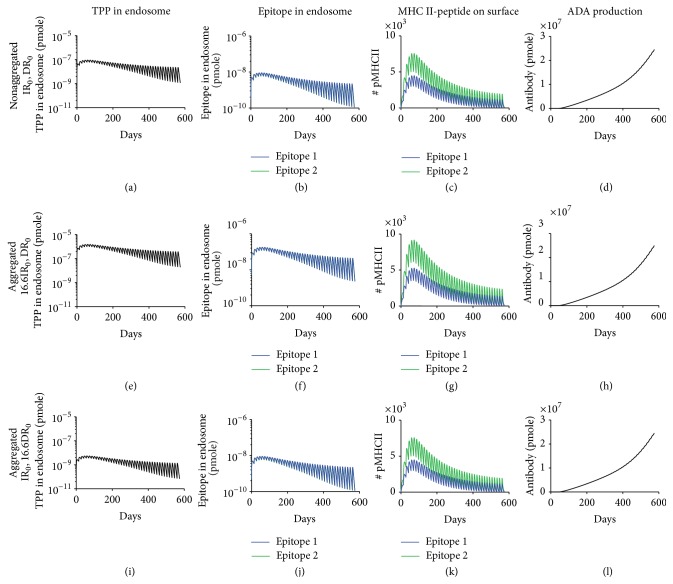
Aggregates could not enhance ADA production through faster antigen internalization or degradation if high affinity epitopes are already present in nonaggregated TPP. Simulated levels of nonaggregated and aggregated TPP in endosome, epitopes in endosome, MHC II-peptide complex on cell surface, and ADA production are shown for ((a)–(d)) original internalization (IR_0_ = 14.4 day^−1^) and degradation (DR_0_ = 17.28 day^−1^) rate for nonaggregated adalimumab [47, 48], ((e)–(h)) 16.6IR_0_ and DR_0_ for hypothetical aggregated form, and ((i)–(l)) IR_0_ and 16.6DR_0_ for hypothetical aggregated form. ADA production has the same definition and dose has the same value as in Figure 4.

**Figure 6:**
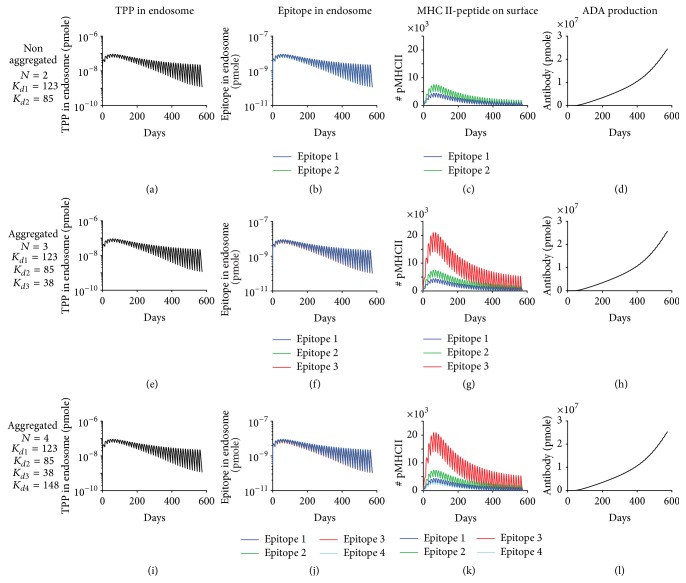
Aggregates could not enhance ADA production by increasing number of epitopes if high affinity epitopes are already present in nonaggregated TPP. Simulated levels of TPP in endosome, epitope in endosome, MHC II-peptide complex on cell surface, and ADA production are shown for ((a)–(d)) original two predicted epitopes for nonaggregated adalimumab [47, 48], ((e)–(h)) three epitopes for hypothetical aggregated form, and ((i)–(l)) four epitopes for hypothetical aggregated form. The predicted dissociation constant (*K*_*d*_, unit: nM) for binding of each epitope to MHC II is indicated. ADA production has the same definition and dose has the same value as in Figure 4.

We previously simulated ADA production induced by adalimumab with an internalization rate of 14.4 day^−1^ (IR_0_), a degradation rate of 17.28 day^−1^ (DR_0_), and two predicted adalimumab epitopes with high binding affinities of 123 and 85 nM to common MHC II allele DRB1^*∗*^04:01 [47]. To model the aggregates' effect on antigen processing, we increased either internalization or degradation rate by 16.6-fold based on a previous study which reported that aggregated mAb resulted in a 16.6-fold increase in total peptides associated with MHC II [30] and then assessed the levels of endosomal aggregates and epitopes, MHC II-peptide complexes on cell surface, and ADA production. As expected, conditional on the parameters and structure of the model simulation, increasing internalization rate by 16.6-fold resulted in a similar fold increase in aggregates internalized into endosome and epitopes generated by its degradation (Figures 5(a)-5(b) and 5(e)-5(f)). Increasing degradation rate by 16.6-fold resulted in the same fold decrease in endosomal aggregates, but the levels of epitopes were unchanged, which suggested that epitope generation was limited by the amount of aggregates internalized and not by the degradation rate (Figures 5(a)-5(b) and 5(i)-5(j)). Moreover, increasing internalization or degradation rate by 16.6-fold did not significantly change the number of MHC II-peptide complexes presented on the surface of APC (Figures 5(c), 5(g), and 5(k)). Aggregates could also impact the FcR binding and potentially affect the antigen uptake [44]. We therefore evaluated a larger range of internalization and degradation rate. Our conclusions were unaffected by larger increases (200-fold) in internalization or degradation rate (data not shown). Consistent with MHC II-peptide complex presentation levels, increasing internalization or degradation rate by 16.6-fold had little impact on final ADA production (Figures 5(d), 5(h), and 5(l)). We next modeled the effect of aggregates on the number of epitopes presented. As expected, including aggregate-induced generation of new epitopes led to the surface presentation of corresponding MHC II-peptide complexes whose levels depend on the binding affinity of epitope to MHC II (Figures 6(a)–6(c), 6(e)–6(g), and 6(i)–6(k)). Surprisingly, if two high affinity epitopes are already present, then the inclusion of new epitopes did not increase ADA production (Figures 6(d), 6(h), and 6(l)). Taken together, these analyses suggest that aggregate-induced high antigen processing and presentation cannot enhance ADA production if high affinity epitopes are already present.

### 3.4. Aggregates Could Enhance ADA Production by Inducing the Presentation of Epitopes with Higher Affinities than Those from Nonaggregated TPP

**Figure 7:**
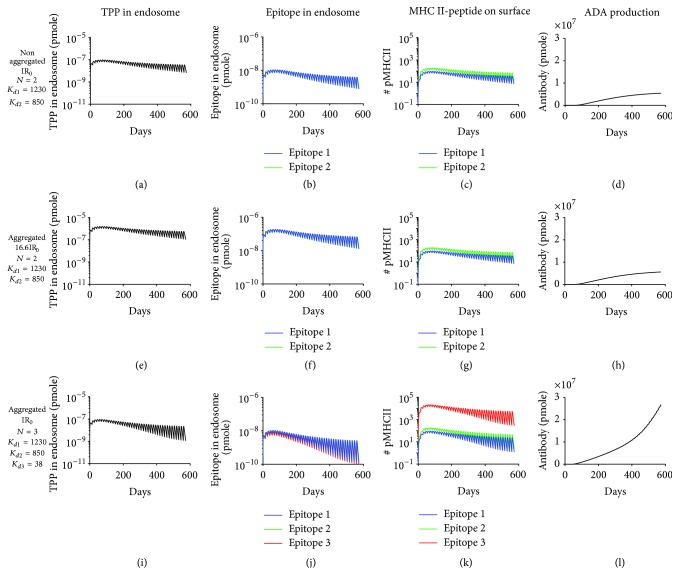
Aggregation could contribute to ADA production by inducing the presentation of high affinity epitopes that may not be present in nonaggregated TPP. Simulated levels of TPP in endosome, epitope in endosome, MHC II-peptide complex on cell surface, and ADA production are shown for ((a)–(d)) original internalization rate (IR_0_) and two low affinity epitopes for hypothetical nonaggregated TPP, ((e)–(h)) 16.6IR_0_ and two low affinity epitopes for hypothetical aggregated form, and ((i)–(l)) IR_0_ and inclusion of a high affinity third epitope for hypothetical aggregated form. The predicted dissociation constant (*K*_*d*_, unit: nM) for binding of each epitope to MHC II is indicated. ADA production has the same definition and dose and IR_0_ have the same values as in Figure 4.

**Figure 8:**
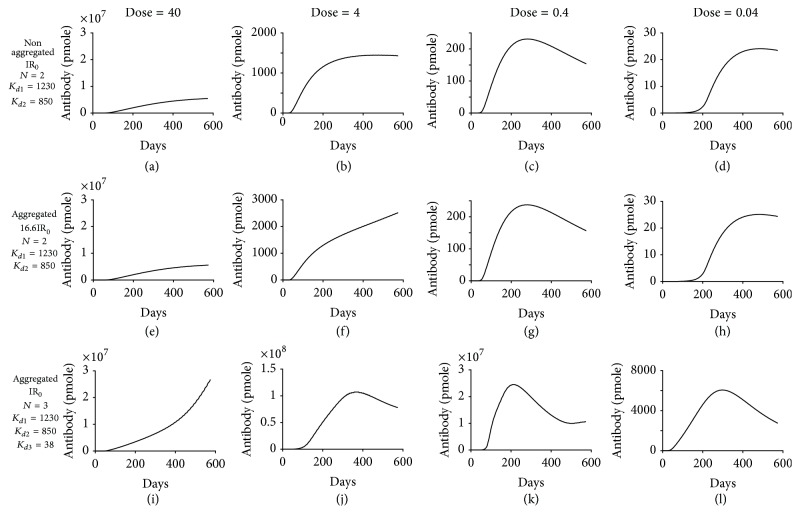
Aggregation could contribute to immunogenicity by inducing the presentation of high affinity epitopes that may not be present in nonaggregated TPP under a variety of drug doses. Simulated ADA production is shown for the same conditions as in Figure 7 for biweekly administered dose of 40 mg ((a), (e), and (i)), 4 mg ((b), (f), and (j)), 0.4 mg ((c), (g), and (k)), and 0.04 mg ((d), (h), and (l)). ADA production has the same definition as in Figure 4.

MHC II-restricted epitopes are generated with *μ*M to nM affinity range [65]. We next evaluated whether aggregate-induced high antigen processing and presentation could increase immunogenicity when nonaggregated TPP present low affinity (*μ*M range) epitopes. We started with 40 mg dose of nonaggregated TPP administered biweekly and two epitopes with *K*_*d*_ of 1230 and 850 nM representing low affinity epitopes of *μ*M range [65, 66] and monitored the number of MHC II-peptide complexes on surface of APC and ADA production (Figures 7(a)–7(d)). We next increased the internalization rate by 16.6-fold to mimic the effect of aggregates and again saw no increase in antigen presentation and ADA production (Figures 7(e)–7(h)). Notably, when aggregates induced the presentation of a high affinity epitope (*K*_*d*_ = 38 nM), ADA production increased by >4-fold (Figure 7(l)) due to enhanced antigen presentation (Figures 7(i)–7(k)). We further evaluated the effect of aggregate-induced high affinity epitopes on ADA production under different dose levels, all of which demonstrated that induction of a high affinity epitope could significantly increase ADA production (compare top and bottom rows in Figure 8), whereas increase in internalization rate had no effect (compare top and middle rows in Figure 8). These computational modeling results indicate that aggregates could contribute to ADA production by inducing the presentation of high affinity epitopes that may not be present in nonaggregated TPP.

## 4. Discussion

In this study, we used mathematical modeling to comprehensively evaluate mechanisms through which aggregates of TPP could contribute to immunogenicity. By modeling the interactions between aggregates and BCRs, we find that aggregates are unlikely to induce T cell-independent antibody responses through BCR cross-linking due to the limited number of STC that could form under physiologically plausible conditions. Thereafter, using our previously developed multiscale, mechanistic mathematical model for the T cell-dependent induction of ADA by TPP, we systematically evaluated the potential roles of aggregates in ADA generation by dissecting the individual steps leading to it. Our analyses indicate that aggregates could contribute to immunogenicity by increasing the danger signal to maturate dendritic cells and activate T cells and/or by inducing the presentation of high affinity epitopes that may not be present in nonaggregated TPP.

TPP could aggregate during manufacturing, storage, handling, or delivery to patients due to agitation, light exposure, temperature elevation, oxidation, pH change, and leaching [12, 17, 23, 24, 29, 30, 56, 67]. Aggregation has been proposed as a strong risk factor for TPP-induced immunogenicity due to its potential role in both T cell-independent and T cell-dependent antibody responses [10, 12, 14, 16, 17]. Several previous studies in mice have demonstrated that for different TPP aggregates induced a stronger ADA production compared with nonaggregated forms [18–21, 25, 27, 28]. However, the mechanisms underlying aggregate-induced ADA production are not clear. A recent study in mice transgenic for human IgG demonstrated that only light-induced oligomers of IgG induced an immune response, which was ablated by the depletion of CD4+ cells [66]. The data from this mouse model are in agreement with the mathematical model in which aggregates induce immune responses in a T cell-dependent manner.

Repetitively arranged epitopes in a paracrystalline structure of viral particles could cross-link BCRs to induce T cell-independent IgM or in some cases IgG3 responses [68–72]. It is expected that aggregates of TPP, potentially resembling the structure of highly repetitive epitopes, could induce T cell-independent antibody responses in a similar way [12, 16, 17]. The model does not directly consider the nature of a polyclonal B cell response, but it is consistent with that. Specifically, multiple epitopes from the aggregates being bound by the BCR can be represented by the differential binding rate constants in the model, and the different number of B cell epitopes on aggregates can be captured by the complex forming between aggregates and various number of BCRs. Surprisingly, by modeling the interactions between aggregates and BCRs, we find that aggregates are unlikely to induce T cell-independent antibody responses because only a few STC can form under physiologically plausible conditions for antigen-specific B cells, antigen dose, and binding affinity (Figure 2(b), left and center panels). This is consistent with previous studies in mice that showed no significant T cell-independent IgG3 antibody response against aggregated recombinant murine growth hormone [28] or anti-TNF*α* murine mAb [23], although IgM production was not evaluated in either case. However, it should be noted that, under conditions of high binding affinity and BCR concentration and appropriate antigen concentrations, significant number of STC could form, with a potential to induce T cell-independent antibody response (Figure 2(b), right panel). High BCR concentration can be achieved through high percentage of antigen-specific B cells for particular TPP or through B cell proliferation due to lowering of activation threshold by cytokines [73], second messenger diacylglycerol [74], costimulatory signal [75], or Bruton's tyrosine kinase [76]. Appropriate antigen concentration can result from specific dosing strategies. Therefore, particular attention should be given while administering TPP to patients in those conditions. Future experiments directly investigating the downstream signaling of BCR cross-linking in the presence of aggregated and nonaggregated TPP and studies evaluating whether T cell-independent IgM is induced in response to aggregates can further elucidate the role of aggregates in T cell-independent ADA production.

T cell-dependent ADA production is thought to be the major pathway through which TPP induce immunogenicity as in the case of IgG1 and IgG4 generated against anti-TNF*α* mAb to treat a variety of inflammatory and autoimmune diseases [7]. Antigen processing and presentation by professional APC, such as dendritic cells, macrophages, and B cells, play a key role in T cell-dependent antibody responses [12]. It has been shown that aggregates could enhance antigen uptake by APC thereby increasing peptides associated with MHC II and could induce dendritic cell maturation and T cell activation [22, 30, 56]. However, human data directly ascribing ADA levels to aggregates are still lacking. In this study, we systematically evaluated whether aggregate-enhanced antigen processing and presentation could increase ADA production. Our computer simulation suggests that the amount of antigenic peptides in endosome is limited by antigen internalization rate, not degradation rate, and the number of MHC II-peptide complexes presented on cell surface is mainly restricted by the binding affinity of epitopes. Our modeling analyses indicate that induction of high affinity epitopes by aggregates that may not be present in nonaggregated TPP and increased danger signal by aggregates to maturate dendritic cells could result in increased ADA production (Figures 4–8). A specifically designed experimental study that examines the binding affinities of peptides to MHC II derived from dendritic cells treated with aggregated or nonaggregated TPP would verify whether aggregates can induce the presentation of high affinity epitopes not present in nonaggregated TPP. In this work, we modeled aggregates-induced danger signal as LPS. However, it should be noted that aggregates have the potential to bind to a variety of pattern recognition receptors as well as FcR. Therefore the kinetics, activation thresholds, and receptors engaged by aggregates are more diverse and complicated than those of LPS and need further investigation.

This work improves our understanding of aggregate-induced immunogenicity and could be utilized to develop prediction and mitigation strategies. Overall, this modeling study suggests that aggregates could enhance immunogenicity; therefore enough attention should be given to reduce aggregation during manufacturing, storage, handling, and administration. In particular, potential high affinity CD4+ T cell epitopes are of great concern because their presentation in nonaggregated TPP will result in high levels of immunogenicity regardless of aggregation. On the other hand, even if they are not presented in nonaggregated TPP, an aggregation-induced presentation will also result in enhanced immunogenicity. Thus, efforts should be made towards experimental identification or* in silico* prediction of high affinity epitopes during immunogenicity assessment, and potential high affinity epitopes should be avoided while designing novel TPP as they carry a strong risk for ADA generation.

Our recently developed mechanistic system-level mathematical model for ADA production is a useful tool to evaluate human immunogenicity against TPP as it incorporates protein-specific antigenic properties and host-specific immunological characteristics, although further experimental validation is needed to increase confidence in ADA predictions [47, 48]. Multiple product- and patient-related risk factors have been proposed to impact immunogenicity of TPP [7, 8, 10–14, 77, 78]. As confidence in its properties increases, this system-level model could potentially be used to design new hypotheses and study other risk factors besides aggregation. For example, though the model is developed for healthy subjects, it can be easily modified to account for the effect of different disease statuses. For example, the profile of ADA generation observed in autoimmune patients [79, 80] can be simulated by including either a lower activation threshold for immune cells [37, 38] or preexisting immunity against TPP [79, 80]. Also, peptide editor HLA-DM plays a key role in MHC II antigen presentation and CD4+ T cell epitope selection by favoring the presentation of peptides with higher kinetic stabilities [65, 81–84]. To evaluate the effect of HLA-DM-mediated epitope selection on ADA production, it could be included in the subcellular module of antigen processing and presentation to select the epitopes presented based on peptide susceptibility to HLA-DM-mediated peptide exchange [84]. Other ADA production impact factors that could be evaluated by this system-level model include time delays between administration, immune cell activation and migration from tissue to lymphoid compartments [42], contraction of effector B cells and T cells [85, 86], effect of immunomodulators through comedication [87], and different antibody isotypes generated by short- and long-lived plasma cells [42, 88, 89]. Therefore, this model could generate new hypotheses about immunogenicity and could be used with experiments to decipher the mechanisms underlying immunogenicity of TPP and develop corresponding mitigation strategies.

## 5. Conclusion

In summary, our computational analyses suggest that aggregates are unlikely to induce T cell-independent antibody responses through BCR cross-linking due to limited formation of STC under physiologically plausible conditions. In contrast, aggregates could contribute to immunogenicity via the T cell-dependent pathway by inducing the presentation of high affinity epitopes that may not be present in nonaggregated TPP and/or by enhancing danger signal to maturate dendritic cells and activate T cells. This study provides novel insights into how aggregates could contribute to overall immunogenicity and suggests novel mechanistic hypotheses eventually suitable for experimental testing.
